# Place of death of people living with Parkinson’s disease: a population-level study in 11 countries

**DOI:** 10.1186/s12904-015-0021-3

**Published:** 2015-05-20

**Authors:** Katrien Moens, Dirk Houttekier, Lieve Van den Block, Richard Harding, Lucas Morin, Stefano Marchetti, Agnes Csikos, Martin Loucka, Wayne A Naylor, Donna M Wilson, Joan Teno, Marylou Cardenas-Turanzas, YongJoo Rhee, Francisco Javier Garcia-Leon, Luc Deliens, Joachim Cohen

**Affiliations:** Department of Palliative Care, King’s College London, Cicely Saunders Institute, Policy & Rehabilitation, Bessemer Road, Denmark Hill, London, SE5 9PJ UK; Vrije Universiteit Brussel (VUB) & Ghent University, End-of-Life Care Research Group, Brussels, Belgium; French National Observatory on End-of-Life Care, Paris, France; Italian National Institute of Statistics, Rome, Italy; University of Pécs Medical School, Pécs, Hungary; Center for Palliative Care, Prague, Czech Republic; Hospice Waikato, Hamilton, New Zealand; University of Alberta, Edmonton, Alberta, Canada; Brown University, Department of Community Health, Rhode Island, USA; The University of Texas, MD Anderson Cancer Center, Houston, TX USA; Dongduk Women’s University, Seoul, Korea; Consejería de Igualdad, Salud y Bienestar Social. Agencia de Evaluación de Tecnologías Sanitarias de Andalucía (AETSA), Sevilla, Spain

**Keywords:** Parkinson’s disease, Place of death, Cross-national comparison, Risk factors, Palliative care

## Abstract

**Background:**

Most people prefer to receive end-of-life care in familiar surroundings rather than in hospital. This study examines variation in place of death for people dying from Parkinson’s disease (PD) across 11 European and non-European countries.

**Methods:**

Using death certificate data of 2008 for Belgium, France, Italy, Hungary, Czech Republic, New Zealand, USA, Canada, Mexico, South Korea and Spain for all deaths with PD as an underlying cause (ICD-10 code: G20) cross-national differences in place of death were examined. Associations between place of death and patient socio-demographic and regional characteristics were evaluated using multivariable binary logistic regression analyses.

**Results:**

The proportion of deaths in hospital ranged from 17% in the USA to 75% in South Korea. Hospital was the most prevalent place of death in France (40%), Hungary (60%) and South Korea; nursing home in New Zealand (71%), Belgium (52%), USA (50%), Canada (48%) and Czech Republic (44%); home in Mexico (73%), Italy (51%) and Spain (46%). The chances of dying in hospital were consistently higher for men (Belgium, France, Italy, USA, Canada), those younger than 80 years (Belgium, France, Italy, USA, Mexico), and those living in areas with a higher provision of hospital beds (Italy, USA).

**Conclusions:**

In several countries a substantial proportion of deaths from PD occurs in hospitals, although this may not be the most optimal place of terminal care and death. The wide variation between countries in the proportion of deaths from PD occurring in hospital indicates a potential for many countries to reduce these proportions.

## Background

With population ageing and possibly other etiological factors the number of people living with Parkinson’s disease is expected to grow substantially in the coming decades [1].

The provision of optimal end-of-life care for people living with Parkinson’s disease is challenging because the disease trajectory is longer and less predictable than other progressive illnesses such as cancer [2]. In advanced stages of Parkinson’s disease, sufferers are also more likely to develop several co-morbidities and complications, such as thrombosis, infections of the lung and urinary tract, and dementia [3,4]. Their progressive decline in function and increasing dependency on others for care often results in people with Parkinson’s disease needing institutional long-term care (LTC) [4].

One of the objectives of palliative care is enabling patients to die where they prefer [5] and ‘place of death’ has been proposed as a quality indicator of palliative care [6]. Most people would prefer to die in familiar surroundings, such as at home or in the nursing home where they live [7]. Although hospitals may be a common place of death, the evidence shows that hospitals often provide suboptimal whole-person care resulting in less favourable outcomes for patients and their relatives [8].

Until now, comparative international place of death research focused on people with Parkinson’s disease has not been reported.

We aim to answer 1) what is the place of death for people dying from Parkinson’s disease across European and non-European countries, and 2) to what extent are demographic, social, residential and health care system factors associated with dying in a hospital in these countries?

## Methods

### Study design

#### Procedures

This study is part of the International Place of Death (IPoD) study, collecting death certificate data for the full population of deaths for a period of one year in several countries [9].

An open call tendering for partners was launched by the principal investigators of the study. Candidate partners were required to negotiate with national health or other authorities to collect all individual death certificate data of 2008 or the nearest available year, including place of death and factors indicated in the literature as relevant predictors of the place of death [10] for inclusion in an international database. The year 2008 was chosen as a reference year because an exploration of all candidate partners learned that, at the time of the data collection (2011–2013), this was the most recent available year in all targeted countries together. Fourteen of 27 candidate countries were able to obtain all necessary permissions to use the national death certificate data and have these integrated into an international database.

#### Population

For this study we used the death certificate data of eleven countries: Belgium, France, Italy, Spain (only Andalusia region), Hungary, Czech Republic, New Zealand, USA, Canada (excluding Quebec province), Mexico and South Korea (N_all deaths_ = 4,925,862). These eleven countries provided data with three or four digit International Classification of Diseases (ICD)-10 codes [11] which allowed us to select deaths with Parkinson’s disease as underlying cause of death (ICD-10 code for Parkinson’s disease: G20). The three countries that were not included provided the underlying cause of death in aggregated categories due to data protection measures.

Some death certificates contain socio demographic information of the deceased, information often supplied by a civil servant of the civil registrar of the municipality of death, for other countries this information is not available through death certificates. However, linkage with other databases (e.g. census data) allowed inclusion of a minimum number of socio demographic and environmental related variables in most countries.

### Measurements

The dependent variable for our study is place of death i.e. home, hospital, LTC-setting, and hospice (only available as a separate category on the death certificate data in New Zealand and the USA), other institution (e.g. institutions for people living with mental disabilities) and other (e.g. public road). The death certificated data of the USA captured free standing hospice inpatient units and not the hospital based palliative care units. To describe the place of death across countries we combined ‘other institution’ and ‘other’ into one category (‘other’). To examine which factors are associated with hospital death within each country we dichotomized hospital versus all other places of death.

As independent variables we used four categories of factors [10]: 1) demographic and social factors: sex, age (<65, 65–79 and 80+) and educational attainment (no formal or elementary, lower secondary, higher secondary, and higher); 2) social support: marital status (unmarried, married, widowed, and divorced/separated); 3) residential factors: the degree of urbanization of the place of residence (strong/very strong, average, weak/rural); and 4) health care system factors: mean number of provided hospital beds within the health care region per 1000 inhabitants and mean number of beds within the health care region in LTC-settings per 1000 inhabitants 65+ years. Educational attainment was not available for France, New Zealand and Canada. Marital status was not available for New Zealand. Urbanization level was not available for the Czech Republic, New Zealand, USA and Mexico. In Canada information concerning the urbanization level was only available for two categories: urban or rural.

For Belgium, France, Italy, Spain, New Zealand, USA, Canada and Mexico the data on mean number of long term care beds provided per 1000 inhabitants aged 65+ and mean number of hospital beds provided per 1000 inhabitants were supplied per health care or administrative region. For Hungary, Czech Republic and South Korea these were provided only for the country as a whole as data protection regulations precluded having data on region of residence.

### Data analysis

To describe the bivariate associations between each independent variable and place of death (hospital death versus all other places of death) we used contingency tables and X^2^ tests (significance level: p ≤ 0.05).

For each country separately, we used a multivariable binary logistic regression model to estimate the factors associated with dying in a hospital. A forward stepwise likelihood ratio selection of factors and covariates method was used to construct the best-fitting but still parsimonious model. In this model some of the categories of the independent variables were combined into two-category variables: age (−80 years vs. 80+), educational attainment (no formal or elementary and lower secondary vs. higher secondary and higher), marital status (unmarried, widowed and divorced/separated vs. married), and urbanization level (average/weak/rural vs. very strong/strong).

We used SPSS version 18.0 for all statistical computations.

### Ethics

The use of anonymised death certificate data did not require research ethics approval. However, the necessary permissions from the data protection agencies were obtained in all countries providing data for the study.

## Results

### Study population

The proportion of people dying from Parkinson’s disease ranged from 0.1% in Czech Republic to 0.8% in Belgium, France, USA and Canada (Table 1). At least half were male, except in South Korea where 56.3% were female. The majority of the people dying from Parkinson’s disease were 80+ years old across all countries, except for South Korea and Hungary where the majority was younger than 80. In all countries, more than 45% were married, while between 36% (in Mexico) and 46% (in South Korea) were widowed.

**Table 1:** **Characteristics of people dying from Parkinson’s disease (N = 34,430)**

**Characteristics**	**Belgium**	**France**	**Italy**	**Spain (Andalusia)**	**Hungary**	**Czech Republic**	**New Zealand**	**USA**	**Canada**	**Mexico**	**South Korea**
N of deaths (proportion on all deaths in %):	837(0.8)	4,599(0.8)	4,034(0.7)	352(0.6)	241(0.2)	92(0.1)	202(0.7)	20,065(0.8)	1,381(0.8)	1,062(0.2)	1,565(0.6)
*Demographic and social factors*											
Sex:											
male	52.0	53.2	50.0	55.4	58.5	58.7	52.5	57.6	56.3	55.7	43.7
female	48.0	46.8	50.0	44.6	41.5	41.3	47.5	42.4	43.7	44.3	56.3
Age:											
<65 years	1.4	1.5	1.6	2.0	2.5^§^	9.8	4.0	2.4	2.2	8.3	7.7
65 to 79 years	36.9	27.0	30.8	35.2	58.1	41.3	31.2	30.0	30.3	40.2	58.1
80+ years	61.6	71.5	67.6	62.8	39.3	48.9	64.9	67.7	67.6	51.5	34.2
Educational attainment:											
no formal or elementary	44.9*	_	80.6	54.6	67.2	37.1	_	7.4	_	78.4	67.6
lower secondary	23.8	_	10.9	28.1	0.4	48.3	_	8.9	_	1.6	_
higher secondary	22.4	_	5.2	13.6	18.1	10.1		43.2		7.5	23.1
higher	8.9	_	3.3	3.8	14.2	4.5	_	40.5	_	12.5	9.3
*Social support*											
Marital status:											
unmarried	5.9	8.8	9.5	8.7	5.8	1.1	_	4.9	6.3	11.2	1.9
married	48.5	46.4	45.7	47.7	51.5	54.3	_	48.4	48.8	51.6	49.6
widowed	40.6	40.8	44.0	42.4	37.3	34.8	_	40.0	40.0	35.5	46.3
divorced/ separated	5.0	3.9	0.9	1.2	5.4	9.8	_	6.7	4.9	1.7	2.3
*Residential factors*											
Urbanization level:											
very strong/strong	57.6	32.8	42.8	55.4	35.7	_	_	_	82.8^†^	_	17.8
average	25.3	32.2	19.5	39.5	37.3	_	_	_	_	_	20.9
weak/rural	17.1	35.0	37.6	5.1	27.0	_	_	_	16.7^†^	_	61.3
*Health care system factors*											
Hospital beds/1000 inhabitants	5.2	6.9	3.4	2.7	7.1^‡^	6.0^‡^	3.1	2.7	3.0	0.3	8.3^‡^
LTC-beds/1000 inhabitants age 65+	70.3	58.4	34.6	17.7	32.7^‡^	28.9^‡^	71.6	41.5	58.1	0.0	17.5^‡^

‘-‘ in cells represents that this information was not available in the country’s death certificate data. Percentages are valid column percentages. *Abbreviation: LTC* Long Term Care.

*For Belgium 41.2% of the cases had missing information for educational attainment. Percentages are valid percentages only.

^†^Within Canada the urbanization level was categorized as ‘urban’ and ‘rural’.

^‡^Data were provided for the country as a whole.

^§^For Hungary the age distribution was provided in different ranges: <60; 60–79; 80 + .

### Place of death

Only in two countries, home was the predominant site of death (Mexico and Italy with 73% and 51%, respectively) (Table 2). Nursing homes were the predominant sites of death in New Zealand (71%), Belgium (52%), and USA (50%). Dying in an acute care hospital varied from 17% in USA to 75% in South Korea. In New Zealand, no people living with Parkinson’s disease died in a hospice; in the USA this was 4.0% (data not shown in Table 2).

**Table 2:** **Place of death of people dying from Parkinson’s disease (N = 34,430)**

	**Belgium**	**France**	**Italy**	**Spain (Andalusia)**	**Hungary**	**Czech Republic**	**New Zealand**	**USA**	**Canada**	**Mexico**	**South Korea**
*Places of death* *											
Hospital	26.9	39.7	31.3	38.1	60.2	39.1	18.8	17.3	42.4	24.1	74.7
Home	20.8	33.4	51.0	46.0	_	16.3	5.9	23.8	5.9	73.0	20.6
LTC-setting	51.7	24.2	13.4	15.1	_	43.5	71.3	49.7	47.6	_	4.5
Other	0.6	2.7	4.2	0.9	39.8	1.1	4.0	5.2	4.1	2.9	0.2

‘-‘ in cells represents that this information was not available in the country’s death certificate data. Percentages are valid column percentages.

*Abbreviation: LTC* Long Term Care.

*USA and New Zealand were the only countries where the death certificate data provided detail on hospice as a place of death. In the USA 4.0% died in a hospice; in New Zealand this was 0.0%.

### Hospital death by patient and ecological characteristics

Men significantly more often than women died in hospital in Belgium, France, Italy, USA and Canada (Table 3). Younger patients more often died in a hospital in Belgium, France, Italy, USA, Canada, and South Korea. Significant differences according to educational attainment were only found in Mexico, with those with a higher education more often dying in a hospital. Married persons more often than non-married persons died in a hospital in Canada and France. In Italy and the USA, hospital deaths were significantly more likely in regions with a higher provision of hospital beds.

**Table 3:** **Proportions of Parkinson’s disease deaths occurring in hospital by patient and ecological characteristics (N = 34,430)**

**Characteristics**	**Belgium**	**France**	**Italy**	**Spain (Andalusia)**	**Hungary**	**Czech Republic**	**New Zealand**	**USA**	**Canada**	**Mexico**	**South Korea**
*Demographic and social factors*											
Sex:											
male	33.1***	46.3***	34.6***	39.0	63.8	38.9	23.6	19.8***	50.0***	23.7	74.1
female	20.1	32.3	28.1	36.9	55.0	39.5	13.5	14.1	32.7	24.6	75.1
Age:											
<65 years	50.0***	49.3***	36.7***	42.9	66.7^§^	44.4	25.0	29.7***	60.0**	23.3	80.0**
65 to 79 years	36.2	47.6	35.9	39.5	60.0^§^	36.8	22.2	19.8	46.9	28.2	71.7
80+ years	20.7	36.6	29.1	37.1	60.0	40.0	16.8	15.8	39.9	21.2	78.5
Educational attainment:											
no formal or elementary	25.8	_	30.7	35.8	55.1	30.3	_	19.6	_	21.5***	73.2
lower secondary	35.9	_	36.6	43.8	100.0	39.5	_	16.9	_	31.3	_
higher secondary	32.7	_	31.7	41.9	64.3	44.4	_	17.2	_	24.0	77.8
higher	27.3	_	28.2	33.3	69.7	75.0	_	17.3	_	38.4	79.7
*Social support*											
Marital status:											
unmarried	24.5	32.9***	30.1	26.7	57.1	0.0	_	20.3***	37.2***	21.1	65.5**
married	31.1	47.0	32.7	42.7	67.7	42.0	_	19.4	50.3	23.1	70.7
widowed	22.7	33.3	29.9	34.2	52.2	40.6	_	14.2	33.6	25.3	79.3
divorced/ separated	21.4	36.7	40.5	75.0	46.2	22.2	_	19.2	46.3	29.4	75.0
*Residential factors*											
Urbanization level:											
very strong/ strong	29.3	39.2	35.3***	37.4	67.4	_	_	_	42.9^†^	_	73.7**
average	24.5	39.5	35.8	38.1	51.1	_	_	_	_	_	82.6
weak/ rural	22.4 --	40.5	24.6	44.4	63.1	_	_	_	39.0^†^	_	72.3
*Health care system factors*											
Hospital beds/ 1000 inhabitants:											
<= median	25	40	23***	40	‡	‡	19.6	16.7*	41	23	‡
> median	29	40	39	35	‡	‡	17.8	18.0	44	25	‡
LTC-beds/ 1000 inhabitants age 65+:											
<= median	27	39	23***	36	‡	‡	24.8	17.3	41	_	‡
> median	27	41	39	40	‡	‡	11.8	17.4	47	_	‡

‘-‘ in cells represents that this information was not available in the country’s death certificate data. Percentages are row percentages. *Abbreviation: LTC* Long Term Care.

*Significant differences between categories within variable at 0.05 level, tested with Fisher Exact Test.; **Significant at 0.01 level ; ***Significant at 0.001 level.

^†^In the Canadian death certificate data the urbanization level was categorized as ‘urban’ and ‘rural’.

‡Data on the region of residence within the country was not provided in the country’s death certificate data and hence the data on health care availability was for the country as a whole.

^§^For Hungary the age distribution was provided in different ranges: <60; 60–79; 80 + .

### Independent factors associated with hospital death

The multivariable binary regression analysis for each country showed that the odds of male patients dying in hospital were higher in Belgium, France, Italy, USA and Canada (Table 4). Being younger than 80 years was associated with higher odds of hospital death in Belgium, France, Italy, USA and Mexico; but with lower odds of dying in hospital in South Korea (odds ratio (OR) = 0.5, 95% confidence interval (CI) = 0.3-0.7). In Mexico, having a higher education degree increased the odds of dying in a hospital (OR = 1.8, 95% CI = 1.3-2.5). In France, Hungary, USA and Canada, married patients had a higher chance of dying in hospital. The Italian people dying from Parkinson’s disease who lived in an urban environment had a higher chance of dying in a hospital (OR = 1.4, 95% CI = 1.2-1.6). Living in an area with a higher provision of hospital beds increased the odds of dying in hospital in Italy (OR = 5.3, 95%CI = 4.0-7.1) and in the USA (OR = 1.1, 95% CI = 1.1-1.2), while living in an area with a higher provision of LTC-beds increased the odds of dying in hospital in France (OR = 1.01, 95% CI = 1.002-1.014).

**Table 4:** **Associated factors with hospital death in people dying from Parkinson’s disease (N = 34,430)**

**Characteristics**	**Belgium**	**France**	**Italy**	**Spain (Andalusia)**	**Hungary**	**Czech Republic**	**New Zealand**	**USA**	**Canada**	**Mexico**	**South Korea**
OR (95% CI)											
*Demographic and social factors*											
Sex:											
Male (Vs. Female)	2.3 (1.5-3.5)	1.5 (1.3-1.7)	1.4 (1.2-1.6)	NS	NS	NS	NS	1.4 (1.3-1.5)	1.7 (1.4-2.2)	NS	NS
Age											
<80 years (Vs. 80+)	1.8 (1.2-2.7)	1.4 (1.2-1.6)	1.4 (1.2-1.7)	NS	NS	NS	NS	1.3 (1.2-1.4)	NS	1.4 (1.1-1.9)	0.8 (0.6-0.98)
Education:											
Higher Secondary or Higher (Vs. lower than higher secondary.)	NS	-	NS	NS	NS	NS	-	NS	-	1.8 (1.3-2.5)	0.6 (0.5-0.8)
*Social support*											
Marital status											
Married (Vs. Not/No longer married)	NS	1.4 (1.2-1.6)	NS	NS	2.0 (1.2-3.4)	NS	-	1.1 (1.02-1.2)	1.5 (1.3-1.9)	NS	0.6 (0.5-0.8)
*Residential factors*											
Urbanization level:											
Very strong/Strong (Vs. average/weak/rural)	NS	NS	1.4 (1.2-1.6)	NS	NS	-	-	-	NS	-	NS
*Health care system factors*											
Hospital beds/1000 inhabitants (Continuous)	NS	NS	5.3 (4.0-7.1)	NS	*	*	NS	1.1 (1.1-1.2)	NS	NS	*
LTC-beds/1000 inhabitants 65+ (Continuous)	NS	1.01 (1.002-1.014)	NS	NS	*	*	0.96 (0.92-0.99)	NS	NS	-	*
Nagelkerke R^2^	0.17	0.05	0.07	0	0.04	0	0	0.02	0.05	0.03	0.03

‘-‘ in cells represents the variable that was not available for this country and hence not included into the multivariable model.

*Abbreviations: OR* Odds Ratios, *CI* Confidence Interval, *NS* Not statistically Significant, *LTC* Long Term Care.

*Data only available for the country as a whole and hence not entered into the multivariable model.

## Discussion

The results of our study show that a substantial proportion of persons living with Parkinson's disease died in hospital in eleven European and non-European countries. However, cross-national variation in place of death is striking.

To our knowledge this is the first cross-national study to investigate place of death and associated factors in people dying from Parkinson’s disease comparing European and non-European countries. The death certificate data that we used were collected with established and stable collection procedures resulting in reliable data, comparable across nations. Death certificate data comprise all deaths and not just a sample, giving sufficient power to model determinants of place of death even in small sub-populations, such as people who died from Parkinson’s disease [12]. Some limitations of this study exist, however. Parkinson’s disease is possibly under-recorded on death certificates, particularly as an underlying cause of death [13]. This introduces bias in the estimation of the total population of people dying with Parkinson’s disease (ie some patients will be recorded as having another underlying cause of death) but produces less of a problem for the particular purpose of our paper to describe where deaths from Parkinson’s disease take place in different countries. The advantage of only including deaths with Parkinson’s disease as an ‘underlying cause of death’ (as opposed to also including those with Parkinson’s disease as a comorbidity) is that a more homogenous group of patients is selected (who were more likely to be in an advanced stage) and that at least the selected patients do not include those who did not clearly died from Parkinson’s disease related symptoms and problems. Another limitation of our data is that some socio demographic variables known to affect place of death are not available [10]. Furthermore, death certificate data do not provide insight beyond patterns in place of death such as ‘quality of dying’ [12]. Nor do they provide direct insight into the preferences, choices, and decision-making from patients, families, health professionals, and policy planners that underlie the statistical patterns. Ideally, the found patterns are, however, a good start for complementary qualitative research aimed at getting a better understanding of these statistical patterns.

In most countries included in our study, a substantial proportion of people suffering from Parkinson’s disease died in hospital. In people living with Parkinson’s disease, LTC provision is possibly a factor explaining variation in place of death. On average, 30-40% of Parkinson’s disease patients develop dementia in an advanced disease stage [14], and having dementia increases the chance of Parkinson’s disease patients being admitted to a LTC-facility [4]. The low proportion of hospital deaths in Belgium and New Zealand may be explained by the higher provision of beds in LTC-settings [15]. South Korea showed the highest percentage of hospital deaths (74.7%) which may be explained by the limited provided LTC-facilities and the Korean LTC social insurance scheme only coming into operation in July 2008 [16]. In France and Canada, countries that also have a relatively high number of LTC-beds, the proportion of hospital deaths was still substantial (39.7% and 42.4% respectively), which in France may be explained by the provision of long-term care in hospitals to some extent [17]. An established LTC-sector in these countries may therefore not be sufficient to balance for the high hospital death proportions observed. LTC-facilities need to have sufficient skilled nursing capacity and a well-integrated palliative care policy to prevent hospitalisations at the end of life [18]. The implementation of advance care planning and advance directives in the policy of LTC-facilities may be another important factor. Evidence from the USA [19] and Belgium [20] has previously shown that written advance directives are associated with less likelihood of terminal hospitalisations. The large cross-national differences in the proportion dying in hospitals also partly have to be understood in the context of health care organizational choices which have in some countries placed a substantial part of long term care or chronic care within the acute hospital setting rather than in separate residential houses or institutions. In Belgium, hospital death in people dying from cancer was shown to be substantially higher than in people dying from Parkinson’s disease, with 61% of deaths from cancer occurring in hospital in 2003 [21]. This may be a result of the higher hospitalization rate in advanced cancer patients, who are more often hospitalized because of acute pain and symptoms. People living with Parkinson’s disease however have a longer disease and care-need progression trajectory and their specific symptoms are more easily dealt with within a LTC setting [2], also showing to be the place of death where a majority of our study population in Belgium died. However, not only the specificity of the disease but also societal norms and practices regarding end-of-life care in people with Parkinson disease influence patterns of their place of death in people with Parkinson disease.

The results of our multivariable binary logistic regression analyses showed that there are demographic and social (support) factors that can be associated with increased odds of dying in hospital in certain countries. Being married increased the odds of death in hospital in France, Hungary and Canada. Possibly, primary health care and palliative home care services in those countries are insufficiently established to support the primary family caregivers in caring for a person living with Parkinson’s disease at the advanced stage of the disease, resulting in unplanned terminal hospitalizations. Being younger than 80 years was another factor for hospital deaths in Belgium, France, Italy, USA and Mexico. Possibly, younger people are less quickly shifted or referred to palliative and LTC-settings. This could result in these younger patients getting more hospital-based care. Being male was a factor for hospital deaths in Belgium, France, Italy, USA and Canada. There could be many reasons for this, such as female persons who live with Parkinson’s disease being more realistic in relation to their prognosis and life expectancy, and them being more communicative concerning their wishes for care and place of death [22].

Home deaths are another option. In Italy, Spain and Mexico, the majority of people living with Parkinson’s disease died at home (51.0%, 46.0% and 73.0% respectively). The high home death proportions in Italy [23] and Spain [24] may be explained by cultural expectations that families take care of their elderly and sick family members. The high percentage of home deaths in Mexico might be explained by limited choice for other places to die because of the limited availability and the limited affordability of other health care services [25]. Larger families may also be important for home deaths, as family caregivers are critical to home deaths [23,24].

Lastly, there is evidence that the development and provision of palliative care vary greatly by country and region [26,27] and are highly likely to influence place of death.

At the national level, the statistical patterns we found regarding country differences in place of death for Parkinson’s disease and factors affecting these statistical patterns can be used as a good starting point for both researchers and policy makers to strive for a better understanding of these differences and development of possible strategies for improvement. Although our data are only sufficient to generate hypotheses regarding place of death patterns, it may be suggested in line with previous research, that communication concerning end-of-life care wishes and place of death preferences should be initiated early in the disease trajectory especially because of people living with Parkinson’s disease having a high risk of developing cognitive impairment with dementia [14]. Advance care planning could be used as a tool in this communication process. At the institutional level, there should also be attention to the development of palliative services in LTC-settings as it is expected that a considerable number of people living with Parkinson’s disease will be cared for in these settings [4]. Efforts are also needed to prevent inappropriate hospital transfers near death for example by providing more training on palliative care and the disease progression and prognosis of people living with Parkinson’s disease to LTC staff [28], providing more guidelines for palliative care, and increasing financial reimbursement for onsite palliative care delivery.

Our findings also call for a better understanding of the general population’s knowledge of Parkinson’s disease and its implications.

## Conclusions

This study found considerable variability in the proportion of deaths from Parkinson’s disease occurring in hospital across eleven countries. Males and younger people who live with Parkinson’s disease appear to be at higher risk for hospital death.

Unless place of death becomes a major focus for quality end-of-life care, we can expect that persons with serious progressive illnesses, such as those who suffer from Parkinson's disease, will continue to need to rely on hospitals for terminal care. We suggest that end-of-life care strategies should take into account the cultural factors that influence preferences for terminal care and place of death, the organization and functioning of health care systems, and the way health care services are provided in order to prevent people with Parkinson’s disease dying in hospitals.
